# Interventions simultaneously promoting social participation and physical activity in community living older adults: A systematic review

**DOI:** 10.3389/fpubh.2022.1048496

**Published:** 2022-12-07

**Authors:** Antonina Tcymbal, Karim Abu-Omar, Verena Hartung, Annalena Bußkamp, Chiara Comito, Christin Rossmann, Diana Meinzinger, Anne Kerstin Reimers

**Affiliations:** ^1^Department of Sport Science and Sport, Friedrich-Alexander-Universität Erlangen-Nürnberg, Erlangen, Germany; ^2^Federal Centre for Health Education (BZgA), Köln, Germany

**Keywords:** older adults, community-based intervention(s), physical activity, social participation, physical activity promotion

## Abstract

**Background:**

In recent years, there has been a global trend toward an increase in life expectancy and the proportion of elderly people among the population. In this regard, it becomes important to promote active and healthy aging. Physical inactivity and social isolation are both risk factors of many chronic illnesses and highly prevalent in older adults. This challenges communities to develop interventions that reduce these risk factors among elderly populations. The main aims of this study were to summarize community-based interventions that aim to simultaneously promote social participation and physical activity in older adults and to examine their effects.

**Methods:**

We performed a systematic review based on the PRISMA standards. Literature searches were conducted in six scientific databases in July 2021. Articles were included if they had an interventional design, focused on older adults living in the community and measured social participation and physical activity as an outcome. The data were summarized narratively due to the heterogeneity of studies and the variety of outcome measures.

**Results:**

Overall, 46 articles published in English were included. The studies were grouped in (1) interventions with main focus on physical activity promotion; (2) social activities that included a physical activity component; (3) health behavior interventions/ health education interventions; (4) multicomponent interventions; (5) environmental interventions. The majority of the reviewed studies reported positive effects of interventions on physical activity and/or social participation. No study reported negative effects. Analysis of quantitative studies showed that multicomponent interventions have great positive effects on both outcomes. In qualitative studies positive effects were found regardless of intervention type.

**Conclusion:**

This review summarizes the evidence about the effects of community-based interventions that aim to promote social participation and physical activity in older adults. Multicomponent interventions seem to be most suitable for simultaneous promotion of physical activity and social participation. However, high variability in measurement methods used to assess both social participation and physical activity in the included studies made it difficult to compare studies and to indicate the most effective.

**Systematic review registration:**

www.crd.york.ac.uk, identifier: PROSPERO [CRD42021268270].

## Introduction

Physical activity and social participation are both vital components of healthy aging. However, many older adults are less physically active (1) and integrated in their social surroundings than adults younger than 65 years (2). Thus, improving our understanding of how to simultaneously increase both physical activity and social participation among adults is important for promoting health in old age. This is especially relevant when considering demographic shifts which will result in the world's population of older people to double by 2050 (3), and with it, the burden of disease among older adults (4).

Regarding physical activity, there are irrefutable health benefits for older people to stay active (5). Based on such evidence, the World Health Organization has issued guidelines on physical activity and sedentary behavior for older people in 2020 (6). These guidelines state that individuals over the age of 65 years should engage in 150–300 min of moderate or 75–150 min of vigorous physical activity. Despite these recommendations, more than 50% of older people (age 80 years and older) worldwide remain physically inactive, with progress in increasing physical activity having largely plateaued in recent years (7).

A review of reviews suggests that interventions conducted among community dwelling older people can result in increases in physical activity (8). Farrance et al. (9) demonstrated that community-based exercise programs can result in long-term adherence rates of 70%. There is also some, albeit limited, evidence that volunteer-lead physical activity interventions can result in positive health outcomes for older people (10). Furthermore, among the same population, eHealth interventions have also shown to be effective in increasing physical activity levels (11). For community dwelling older adults receiving home care, however, the results of interventions to increase physical activity remain inconclusive (12). Altogether, these findings indicate the potential of community-based interventions for increasing physical activity of older adults.

In addition to physical activity, social participation also plays an important role in the health of older individuals. Currently there are overlapping definitions and operationalizations for the concepts of social participation, social capital, social support. Levasseur et al. reviewed and analyzed definitions of social participation within aging literature and suggested defining social participation as a “person's involvement in activities that provide interaction with others in society or the community” (13). Based on this definition, social capital and social support can be considered as consequences of successful social participation, while the absence of social participation can lead to feelings of isolation and loneliness.

Studies have demonstrated that being less socially isolated (14) and belonging to social groups (15) results in important health benefits. In this regard, Steffens et al. reports the effects of a feeling of belonging on mortality as being similar to the effects of physically activity (15). Face-to-face interactions have also been positively linked to the mental health of older adults (16).

Regarding interventions aimed at decreasing feelings of loneliness and increasing social participation, several reviews have provided evidence of their effectiveness. Nevertheless, the results reported in these reviews are inconclusive: Dickens et al. (17) reported that most interventions targeted at reducing loneliness among older people report at least one positive outcome. In 2005, a review by Cattan et al. revealed that social and educational interventions are suitable to decrease feelings of loneliness among older people (18). A review of interventions to increase social capital by Coll-Planas et al. (19), however, concluded that similar interventions did not reduce feelings of loneliness among older people, although high-quality trials showed positive effects on mental health within this target group. In contrast, Franck et al. (20) reported that three out of five trials were able to reduce loneliness among older adults receiving home care.

Social participation and physical activity among older adults may mutually reinforce each other. A review performed by Lindsay Smith et al. (21), shows that older adults who report receiving more social support (from family members) report higher levels of leisure-time physical activity than their counterparts receiving less support. Pels and Kleinert (22) concluded from their review that physical activity can contribute to a reduction in feelings of loneliness among older adults.

However, ways to simultaneously promote social participation and physical activity among this population group are still unclear. Until now, no systematic review has summarized the literature on interventions aiming to promote both simultaneously. It is currently unclear which types of interventions would optimize the effects of increased social participation and physical activity among older people. In addition, it is uncertain what types of interventions communities should offer to optimize these effects among community-dwelling older adults.

This review therefore sets out to investigate the effects of interventions aiming to simultaneously promote physical activity and social participation among community dwelling older adults. It focuses on studies that have been conducted within real-world community settings, to investigate the effectiveness of these interventions, and thus increase the external validity of the review's findings.

## Methods

### Study design and background

This review was commissioned by the Federal Centre for Health Education (Bundeszentrale für gesundheitliche Aufklärung; BZgA), Germany. The results of the review are intended to support the participatory project “Aging in Balance” (“Älter werden in Balance”; https://www.aelter-werden-in-balance.de). In the project, researchers and project officers from communities collaborate to establish structures for health and physical activity promotion for older people. It is the intention of this project to improve service offers for citizens and exchange good practice in this field. The results of this review are meant to provide guidance to communities on which services they should offer to increase social participation and physical activity among older people. Methods used to conduct this review were aligned to reach this objective by focusing on trials that were able to investigate effectiveness (by conducting the trial in real-world settings) rather than efficacy (by doing randomized controlled trials conducted in laboratory or field setting) (23).

This review was conducted according to the PRISMA (Preferred Reporting Items for Systematic reviews and Meta-Analyses) statement guidelines (24). The protocol was registered in the International Prospective Register of Systematic Reviews (PROSPERO) (www.crd.york.ac.uk) on August 18, 2021, with the registration number CRD42021268270.

### Eligibility criteria

Inclusion and exclusion criteria were formulated based on the PICO approach (25). Only articles from peer-reviewed journals published in English or German that fulfilled the criteria described in the Table 1 were eligible.

**Table 1:** Eligibility criteria.

	**Inclusion criteria**	**Exclusion criteria**
P–population	Study should include only older adults (the average age of the sample should be above 65 years) or in mixed age populations results for older adults are reported separately. Participants were freely living in the community (either at home or in places of residence that, on the whole, do not provide residential health related care or rehabilitative services).	Participants lived in places that provide residential health related care or rehabilitative services (e.g., nursing homes).
I–intervention	All kinds of interventions that simultaneously promote social participation and physical activity by bringing at least two people together in public spaces.	Intervention did not take place in public spaces. Intervention focused only on patients with specific disease, or carried out as part of rehabilitation, or performed by medical staff. Intervention included only instructor/therapist and one participant.
C–comparison	No inclusion criteria.	No exclusion criteria.
O–outcome	Study included subjective or objective measurement of social participation (e.g., loneliness, social isolation, social support, participants' opinions of changes in their social participation during intervention). Study provided exercise classes that increased overall time of physical activity or included subjective or objective measurement of physical activity or any kind of outcome that constitutes a measure of physical performance that is sought to improve by PA/exercise (e.g., strength, mobility, participants' opinions on changes in their PA level during intervention).	Study did not measure outcomes of social participation. Study that did not provide additional physical activity and did not measure physical activity or any kind of outcome that constitutes a measure of physical performance that is sought to improve by PA/exercise (e.g., strength, mobility).
S, study type	Intervention quantitative and quantitative studies (e.g., effectiveness trials undertaken in real-world implementation settings, quasi-experimental studies, post-intervention).	Study had a cross-sectional design.

### Information sources and search strategy

A systematic search was performed on PubMed, CINAHL, SPORTDiscus, PsycINFO, Scopus, and Web of Knowledge in July 2021. By discussion with the research team members and project officers from communities of the project “Aging in Balance”, a comprehensive search strategy was developed using the PICO approach (25) with a combination of keywords in the categories study sample, intervention, and outcomes.

The search formula was as follows:

(“old people” OR “older people” OR elderly OR elders OR aging OR aging OR “old men” OR “old women” OR “older persons” OR “older adults” OR seniors)

AND (“physical activity” OR “physical activities” OR sport OR training OR exercise OR exercises OR fitness OR stretching OR flexibility OR strength OR resistance OR balance OR endurance OR aerobic OR mobility OR walking OR cycling OR yoga OR pilates OR dancing OR swimming OR jogging OR hiking OR “tai chi” OR taichi OR taijiquan OR gardening OR “qi gong” OR qigong)

AND (Intervention OR programme OR program OR trial)

AND (“social interaction” OR “social interactions” OR “social participation” OR “social support” OR “social network” OR “social networks” OR “social isolation” OR “social activity” OR “social activities” OR “social engagement” OR “social involvement” OR “social inclusion” OR “social life” OR loneliness OR “interpersonal communication” OR “interpersonal interaction” OR “interpersonal interactions” OR “interpersonal relationship” OR “interpersonal relationships” OR “community involvement” OR “community participation” OR “community life”).

### Study selection

Two reviewers independently screened and selected relevant articles using the Covidence systematic review software (Veritas Health Innovation, Melbourne, Australia; www.covidence.org). The first stage consisted of screening titles and abstracts. Afterwards, full texts of potentially relevant articles were reviewed. If the title and/ or abstract indicated that the study fulfilled the eligibility criteria or did not provide sufficient information, both reviewers screened the full texts for eligibility. When necessary, Supplementary material were also assessed for additional information. Disagreements between the reviewers were discussed in the research team until a consensus was reached.

### Data extraction

Data of included studies were extracted and summarized by one researcher, with verification by another reviewer, in order to reduce bias and error. Extraction included the following items: general study information (authors, year of publication, country), intervention description (name, type, duration, frequency, length of session, description of control conditions), sample characteristics (age, special conditions, functional status, activity status, language skills, sample size, mean age, proportion of females), type of collected data (quantitative, qualitative or mixed), quantitative (questionnaire, test, etc.) and/or qualitative (interview, focus groups) methods of measuring social participation and physical activity/fitness, results on effects of interventions on social participation and physical activity/fitness level.

### Risk of bias assessment

The methodological quality of each study was assessed with the QualSyst tool- developed by the Alberta Heritage Foundation for Medical Research (26). This tool was chosen because it assists in the evaluation of intervention studies with different designs, including both qualitative and quantitative studies. Quantitative studies were evaluated according to the following 14 criteria: objective, study design, method of subject/comparison group selection, subject characteristics, intervention allocation, blinding, outcome measure definition, exposure measure definition, sample size, analytic methods, estimate of variance, control for confounding, reporting results and conclusions. For qualitative studies the tool provided the following ten criteria: objective, study design, context, connection to theoretical framework, sampling strategy, data collection, data analysis, verification procedure, conclusions, and reflexivity. Depending on the degree to which the specific criteria were met, each item was scored as “yes” = 2, “partial” = 1, or “no” = 0. If an item was not applicable to the study it was marked “N/A” and excluded from the summary score. The summary score (range: 0–1) indicates the risk of bias, with a higher score indicating higher quality and thus less risk of bias.

### Data synthesis and analyses

Before data analyses, the included interventions were grouped into five different intervention types that were defined based on a discussion with project officers from communities of the project “Aging in Balance”: (1) interventions which primarily focused on physical activity promotion; (2) social activities that included physical activity; (3) health education interventions; (4) environmental interventions; (5) multicomponent interventions. The criteria for classification are presented in Table 2. Associations between interventions and changes in social participation and physical activity/fitness level were analyzed for each type.

**Table 2:** Definition of intervention types.

**Type**	**Description**
1) Physical activity/exercise interventions	Interventions with the main focus on offering participants physical activity or exercise. Increased social participation might have been an additional outcome, however interventions did not provide any additional social activities.
2) Social activity interventions	Interventions provided some types of social activities which naturally included some physical activity, but participants did not receive any direct instructions to increase physical activity/exercises.
3) Health education interventions	Interventions included only lectures or consultations on how to improve health by increasing physical activity level and social participation. No exercise or social activities were offered to participants.
4) Environmental interventions	Interventions consisted only of changes in the built environment in a way that it can increase physical activity and social participation for the residents of a community.
5) Multicomponent interventions	Interventions which combined two or more components from the interventions described above. For example, exercise sessions were combined with additional social activities.

The level of evidence within each type of interventions was summarized narratively due to the heterogeneity of studies and the variety of intervention contents and outcome measures, which prevented a quantitative meta-analysis. For the quantitative studies, we used an approach of summarizing results from studies with different designs that has been suggested by Sallis et al. (27) and also been used by Lindsay Smith et.al (21) in a systematic review with a similar topic. Using this approach, each study was rated as + or –, depending on if a statistically significant positive or negative effect of an intervention was found, and as 0 if no statistically significant associations were found. Ratings were assigned separately for effects on social participation and physical activity/fitness level. Overall ratings for quantitative studies in each category were calculated as “0” (No association; 0–33% of the findings supported the association), “?” (indeterminate association; 34–59% of the findings supported the positive or negative association), “+” or “–” (positive or negative association; 60–100% of the findings supported the association).

## Results

### Study selection process

**Figure 1:**
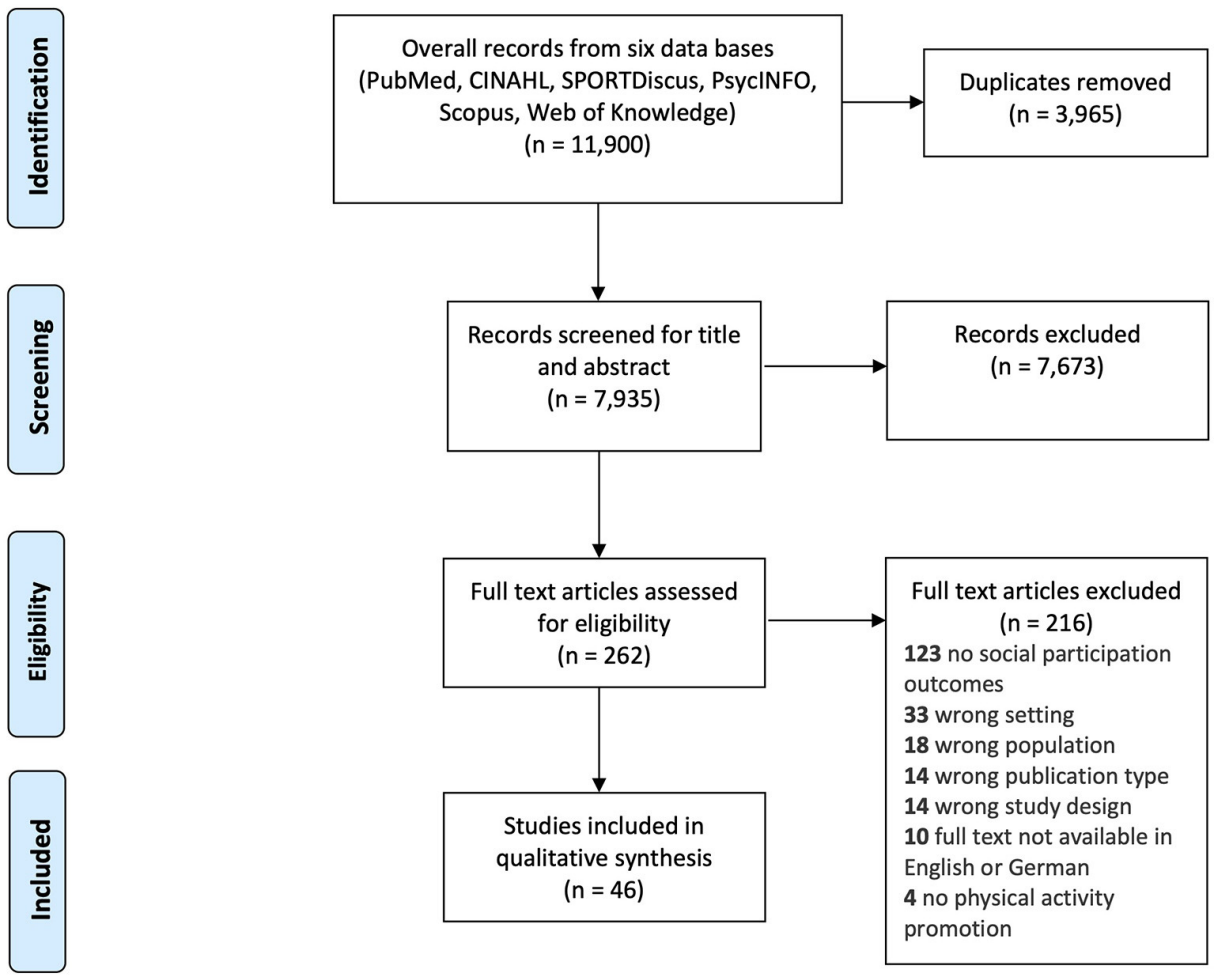
PRISMA flow-diagram of the study selection process.

Electronic searches across the six databases identified 11,900 records, 7,935 remained after removing duplicates. The titles and abstracts were assessed for relevance based on eligibility criteria, resulting in 262 papers retrieved for full text review. After the full text screening, 56 papers met the inclusion criteria. Of these articles, 46 were written in English or German (languages spoken by at least two members of the research team) and included in the analysis. The selection process is presented in a PRISMA flow-diagram (see Figure 1). Characteristics of the included studies are shown as a structured table (see Table 3) and as a narrative summary (see Appendix 1).

**Table 3:** Studies overview.

**References**	**Country**	**Intervention**	**Number of participants**	**Gender composition**	**Data structure**	**Methods of measuring social participation**	**Methods of measuring PA/fitness level**	**Effect on social participation**	**Effect on PA/fitness**	**Quality (quant./qual.)**.
**Physical activity promotion interventions**										
Arnett et al. (36)	USA	PA program to reduce falling risk	120	79.2% female	Mixed	Open-ended survey	Functional fitness tests	Yes	Yes	0.91/0.65
Barbosa et al. (53)	Brazil	Aquatic training program	28	89.3% female	Quantitative	Self-reported questionnaire	–	No	-	0.59
Barraagan (38)	USA	Fitness program	55	Only females	Qualitative	Focus group	–	Yes	–	0.85
Bidonde et al. (35)	Canada	Fitness program	9	Only females	Qualitative	Semi-structured interview	Semi-structured interview	Yes	Yes	0.90
Brustio et al. (54)	Italy	Dance training program	163	75.5% female	Quantitative	Self-reported questionnaire	Functional fitness tests	Yes	Yes	0.77
Carrapatoso et al. (40)	Portugal	Walking	19	84.2% female	Mixed	Semi-structured interview	• Functional fitness tests • Self-reported questionnaire • Semi-structured interview	Yes	Yes	0.68/0.85
Cedergren et al. (55)	USA	Volleyball program	222	79.7% female	Quantitative	Self-reported questionnaire	–	Yes	–	0.91
Chan et al. (56)	China	Tai Chi	48	76% female	Quantitative	Self-reported questionnaire	–	Yes	–	0.88
Dionigi (37)	Australia	Resistance training	10	60% female	Qualitative	Interview	Interview	Yes	Yes	0.70
Ehlers et al. (57)	USA	Dance, or strength/stretching/ stability, or walking, or walking plus (with nutrition supplements)	247	68.4% female	Quantitative	Self-reported questionnaire	–	Yes	–	0.88
Figureira et al. (58)	Brazil	PA program (walking, hydrogymnastics, strengthening, and stretching)	70	62.5% female	Quantitative	Self-reported questionnaire	–	Yes	–	0.54
Frei et al. (59)	Switzerland	Walking groups	29	75.9% female	Mixed	• Self-reported questionnaire. • Structured interview	• Accelerometer • Functional fitness tests. • Structured interview	No	Yes	0.86/0.90
Gomeñuka et al. (60)	Brazil	Nordic walking and free walking	1	72.7% female	Quantitative	Self-reported questionnaire	Functional fitness tests	Yes	Yes	0.92
Kohut et al. (61)	USA	Cardiovascular exercise or strength/flexibility/balance exercise	87	65% female	Quantitative	Self-reported questionnaire	Functional fitness tests	No	Yes	0.88
Komatsu et al. (39)	Japan	Low intensive exercise program	26	57.7% female	Qualitative	Focus group	Focus group	Yes	Yes	0.95
Liu and Tsui (62)	China	Tai Chi	122	87% female	Quantitative	Self-reported questionnaire	• Functional fitness tests. • Self-reported time spent on exercise	No	No	0.92
Maki et al. (63)	Japan	Walking groups	150	70.7% female	Quantitative	Self-reported questionnaire	• Functional fitness tests. • Pedometer	No	Yes	0.77
McAuley et al. (64)	USA	Walking or stretching	174	71.8% female	Quantitative	Self-reported questionnaire	–	Yes	–	0.88
Streber et al. (65)	Germany	Multimodal PA program	87	78.2% female	Quantitative	Self-reported questionnaire	Pedometer	No	No	0.73
Wang (66)	USA	Yoga	18	88.9% female	Quantitative	Self-reported questionnaire	Functional fitness tests.	No	No	0.50
Wickman et al. (32)	Denmark	Floorball	37	only males	Qualitative	Semi-structured interview	Semi-structured interview	Yes	Yes	0.55
**Social activities that included a physical activity component**										
Austin et al. (30)	USA	Community gardening	6	50% female	Quantitative	Self-reported questionnaire	Functional fitness tests	Yes	No	0.41
Boyes (41)	New Zeeland	Outdoor adventure activities	80	63% female	Qualitative	Semi-structured interview	Semi-structured interview	Yes	Yes	0.70
Vadineia da Silva et al. (42)	Brazil	Dance evenings	12	66.7% female	Qualitative	Interview	Interview	Yes	Yes	0.70
Gagliardi et al. (67)	Italy	Social farming	73	63% female	Quantitative	Self-reported questionnaire	Self-reported time spent on exercise	Yes	No	0.80
Gagliardi et al. (33)	Italy	Environmental volunteering and social activities in city parks	19	42.1% female	Mixed	Self-reported questionnaire. Interview	• Self-reported questionnaire • Interview	Yes	Yes	0.75/0.85
Johnson et al. (68)	USA	Choir program	390	76.4% female	Quantitative	Self-reported questionnaire	Functional fitness tests	Yes	No	0.83
**Health behavior interventions/ health education interventions**										
Franke et al. (29)	Canada	Health promotion program	452	77% female	Mixed	Self-reported questionnaire. Qualitative survey	Self-reported PA questionnaire	Yes	Yes	0.86/0.85
McKay et al. (28)	Canada	Health promotion program	458	77% female	Quantitative	Self-reported questionnaire	Self-reported PA questionnaire	Yes	Yes	0.91
Mendoza-Ruvalcaba and Fernández-Ballesteros (43)	Mexico	Health education	76	only females	Quantitative	Self-reported questionnaire	Self-reported PA questionnaire	No	No	0.62
**Environmental interventions**										
Schmidt et al. (34)	Denmark	Renovation of neighborhood open spaces	10	74.2% female	Mixed	• Observing number of people engaged in social interaction. • Interview	Observing number of people engaged in PA	yes	yes	0.64/0.85
**Multicomponent interventions**										
Ahn et al. (69)	South Korea	Health education + exercise + social activities	33	Only females	Quantitative	Self-reported questionnaire	Functional fitness tests	Yes	Yes	0.77
Cardenas et al. (70)	USA	Sport + social activities	444	52% female	Quantitative	Self-reported questionnaire	Self-reported questionnaire	Yes	Yes	0.91
Hopman-Rock and Westhoff (71)	Netherlands	Health education + exercise	390	82% female	Quantitative	Self-reported questionnaire	Self-reported questionnaire	Yes	Yes	0.88
Huang et al. (72)	Taiwan	Tai Chi + cognitive-behavioral intervention	186	58.6% female	Quantitative	Self-reported questionnaire	Functional fitness tests	Yes	Yes	0.92
Hwang et al. (73)	Canada	Health education + fitness program to reduce falls + walking groups + socialization activities	16	94% female	Qualitative	Semi-structured interview	Semi-structured interview	Yes	Yes	0.90
Kamegaya et al. (74)	Japan	Exercise + leisure social activities	52	90.4% female	Quantitative	Self-reported questionnaire	Functional fitness tests	No	No	0.77
Kim et al. (75)	South Korea	Health education + exercise + group activities	39	89.7% female	Quantitative	Self-reported questionnaire	Functional fitness tests	Yes	Yes	0.69
McMahon et al. (76)	USA	Fall-reducing exercise + motivational support	30	93.3% female	Quantitative	Self-reported questionnaire	• Self-reported questionnaire • Accelerometer • Functional fitness tests	Yes	Yes	0.77
McNamara et al. (77)	Australia	Exercise + social activities	21	71% female	Mixed	Interview	Functional fitness test. Interview	Yes	Yes	0.55/0.60
Merchant et al. (78)	Singapore	PA + cognitive exercises + social activities	197	75% female	Quantitative	Self-reported questionnaire	Functional fitness tests	Yes	Yes	0.46
Ren et al. (79)	China	Health education + psychological intervention + Taijiquan exercise	121	N/A	Quantitative	Self-reported questionnaire	–	Yes	–	0.77
Seino et al. (31)	Japan	Exercise + social activities + improving community environment	11,701	51.4% female	Quantitative	Self-reported questionnaire	• Self-reported questionnaire • Functional fitness tests	No	No	0.79
Shvedko et al. (80)	UK	Walks + health education + social interaction	25	56% female	Qualitative	Focus group	Focus group	Yes	Yes	0.80
Yamamoto et al. (81)	Japan	Health education + exercise	31	56% female	Quantitative	Self-reported questionnaire	Functional fitness tests	Yes	Yes	0.75
Yeom and Fleury (82)	USA	Exercise + social support + education + motivational support	64	76.6% female	Quantitative	Self-reported questionnaire	• Self-reported questionnaire • Functional fitness tests	Yes	Yes	0.77

–, No outcome of the category was measured; quant., quantitative; qual., qualitative; PA, physical activity.

### Characteristics of included studies

The 46 papers in this review included reports from 45 studies [McKay et al. and Franke et al. analyzed the same data with different methods (28, 29)]. The included articles were published between 2002 and 2021. Sixteen studies were conducted in North American countries, 12 in Asian countries, ten in European countries, four in Brazil, two in Australia, and one in New Zeeland. There was a total of 16,285 participants across 45 studies, ranging from six (30) to 11,701 (31) per study. The majority of participants were females. There were four studies which consisted solely of female participants, and in 18 studies females made up 75% or more of the sample. Meanwhile, just one intervention was designed exclusively for men (32) and only one further study consisted of more men than women (33). This review included 29 quantitative studies, nine qualitative studies, and seven mixed-methods studies. The way social participation and physical activity were measured and analyzed varied widely between studies.

### Measurements of social participation

Out of the studies which used quantitative methods, 32 applied different self-reported questionnaires to assess post-intervention changes in social participation. In one study, researchers counted the number of participants engaged in social interaction before and after changing the built environment (34). From studies using qualitative methods, nine performed interviews and three conducted focus groups to let participants describe their experiences and perceived changes in social participation during the interventions.

### Measurements of physical activity/fitness

The most common methods of measuring a change in physical activity/fitness level, among the studies which employed quantitative methods, were functional fitness tests (21 studies) and self-reported physical activity questionaries (12 studies). Two studies used accelerometers to objectively assess time spent on physical activity and two studies used pedometers to objectively measure daily steps. Nine studies used interviews and two organized focus groups to gather participants experiences with changes in their physical activity/fitness level. Eight studies engaged participants in extra physical activity without measuring changes in physical activity levels.

### Risk of bias assessment

The average quality score of included studies was 0.77 (0.76 for quantitative studies and 0.78 for qualitative studies) with a range of 0.41–0.95. In most of the quantitative studies, the research question, objective, analysis, and results were sufficiently described and the study design was appropriate. The main sources of bias were related to randomization, blinding of investigators, confounding, and subject selection. The quality of some quantitative studies suffered from poor sample representativeness or lack of a control group. In all qualitative studies, the context of study was well-described, and the conclusions were supported by results. The research question, design, and connection to a theoretical framework were also sufficiently presented in the majority of the studies. The main sources of bias for qualitative studies were related to sampling strategy and absence of reflexivity on how researcher's personal characteristics and methods could have impacted obtained data. Quality scores of the individual studies are reported in Table 3.

### Effects of community-based interventions on social participation and physical activity outcomes

The majority of the reviewed quantitative studies reported interventions having a statistically significant positive effect on social participation (23 studies) and physical activity (18 studies). All qualitative studies showed positive effects on perceived social participation and on the physical activity/fitness levels of the participants. There were no studies which reported negative effects.

The studies were grouped as (1) interventions which primarily focused on physical activity promotion; (2) social activities that included physical activity; (3) health education interventions; (4) environmental interventions; (5) multicomponent interventions. The results of quantitative studies by intervention types are presented in the Table 4.

**Table 4:** Summary of quantitative studies results.

**Outcomes**	**Associations**	**Studies (first author, year, country)**										**Summary score**
**PA/exercise interventions**												
Social participation	Mostly positive*	Cedergren, 2007, USA	Brustio, 2018, Italy	Figureira, 2012, Brazil	Gomeñuka, 2019, Brazil	Chan, 2017, China	Ehlers, 2017, US	McAuley, 2000, USA	Carrapatosoa, 2017, Portugal			? (53.3% positive associations)
	Mostly no association	Streber, 2017, Germany	Frei, 2019, Switzerland	Liu, 2014, China	Kohut, 2006, US	Maki, 2012, Japan	Wang, 2010, USA	Barbosa, 2018, Brazil				
PA/fitness	Mostly positive	Frei, 2019, Switzerland	Carrapatosoa, 2017, Portugal	Kohut, 2006, US	Brustio, 2018, Italy	Maki, 2012, Japan	Gomeñuka, 2019, Brazil	Arnett, 2019, US				+ (70% positive associations)
	Mostly no association	Streber, 2017, Germany	Liu, 2014, China	Wang, 2010, USA								
**Social activities that included PA component**												
Social participation	Mostly positive	Johnson, 2018, US	Gagliardi, 2018, Italy	Austin, 2006, USA								+ (75% positive associations)
	Mostly no association	Gagliardi, 2020, Italy										
PA/fitness	Positive	Gagliardi, 2020, Italy										0 (25% positive associations)
	Mostly no association	Johnson, 2018, US	Gagliardi, 2018, Italy	Austin, 2006, USA									
**Health behavior intervention/Health education (lectures, counseling)**												
Social participation	Mostly positive	McKay, 2018, Canada										? (50% positive associations)
	Mostly no association	Mendoza-Ruvalcaba, 2016, Mexico										
PA/fitness	Mostly positive	McKay, 2018, Canada										? (50% positive associations)
	Mostly no association	Mendoza-Ruvalcaba,2016, Mexico										
**Multicomponent**												
Social participation	Mostly positive	Hopman-Rock, 2002, Netherlands	Ahn, 2014, South Korea	Kim, 2020, South	Cardenas, 2009, US	Ren, 2021, China	Huang, 2011, Taiwan	Yamamoto, 2020, Japan	Yeom, 2014, US	McMahon, 2016, US	Merchant, 2021, Singapore	+ (83.3% positive associations)
	Mostly no association	Seino, 2021, Japan	Kamegaya, 2013, Japan									
PA/fitness	Mostly positive	Hopman-Rock, 2002, Netherlands	Ahn, 2014, South Korea	Kim, 2020, South	Cardenas, 2009, US	Huang, 2011, Taiwan	Yamamoto, 2020, Japan	Yeom, 2014, US	McMahon, 2016, US	Merchant, 2021, Singapore		+ (81.8% positive associations)
	Mostly no associateon	Seino, 2021, Japan	Kamegaya, 2013, Japan									

* The associations were considered as positive only if reported results were statistically significant.

PA, physical activity.

0, No association (0–33% of the findings supported the association); ?, indeterminate association (34–59% of the findings supported the positive or negative association); +, positive association (60–100% of the findings supported the association) [cut-offs were suggested in (27)].

### Interventions focused on physical activity promotion

Of the included studies, 21 described interventions which focused primarily on providing different types of physical activity, and also measured intervention effects on social participation among older individuals. Studies included a total of 1,722 participants. Most of the studies (13 studies) used quantitative methods, five used qualitative methods, and three used mixed methods. During the interventions, participants performed different types of physical activity including walking, dancing, tai chi, fall prevention training, aquatic training, volleyball, and others (see Table 3).

Positive effects of the physical activity interventions on physical activity and fitness outcomes were observed and reported in 70% of the quantitative studies (see Table 4). The effects of the interventions on social participation, however, were not as obvious (53.3% of quantitative studies reported positive associations). In the qualitative studies, participants reported that involvement in the programs gave them the opportunity to get out and be among other people with similar experiences (32, 35), feel more social engagement and social support (36, 37), be more socially connected (38, 39), and expand their communication beyond the group exercise (39). In the participants' opinion, physical activity programs also helped them to improve their physical performance in everyday life (e.g., strength, endurance, balance, coordination) (32, 35, 37, 39, 40).

### Social activities that included a physical activity component

Six of the reviewed studies, including 580 participants, presented the results of interventions which focused on performing different social activities with a physical activity component. These activities included social farming, community gardening, a choir program, dance evenings, outdoor adventure activities, environmental volunteering, and social activities in city parks.

From the four studies that used quantitative methods, three (75%) reported positive effects of the interventions on social participation outcomes; only one study, however, reported positive effects on physical activity/fitness outcomes (33). Qualitative analysis showed that participants noticed improvements in their social participation and felt that the interventions helped them to become more physically active (33, 41, 42).

### Health behavior interventions/health education interventions

Two studies, including a total of 534 participants, examined interventions that promoted physical activity and social participation solely through lectures or consultation. McKay et al. and Franke et al. reported the results of the health promotion program “Choose to Move”, which included consultations with active coaches, motivational meetings, and telephone support (28, 29). Mendoza-Ruvalcaba et al. described the results of “the Vital Aging” program to promote active aging through teaching basic knowledge about aging, and promoting healthy lifestyles (43). McKay et al. and Mendoza-Ruvalcaba et al. collected quantitative data and Franke et al. used a mixed methods approach. The results showed that the effects of health education interventions on social participation and physical activity/fitness outcomes are rather unclear. While the “Choose to Move” program showed positive effects on both social participation and physical activity, the “Vital Aging” program had no effect on either of these outcomes.

### Environmental interventions

Schmidt et al. presented the results of the environmental intervention “Move the Neighborhood” a program based on a participatory research approach, which aimed to increase the use of neighborhood open spaces to promote active living through social interaction and physical activity (34). Together with landscape architects, local older adults explored and developed ideas on how to improve neighborhood open spaces. Renovations were later implemented based on the results of this collaboration. To evaluate the effects of renovations on older adults, authors used a mixed methods approach. They gathered data on the amount of older people engaged in social interaction and physical activity in renovated areas and conducted qualitative interviews with older people to see how these renovations affected social interaction. The environmental intervention study showed that renovating the neighborhood open spaces had some positive effects on social participation and physical activity.

### Multicomponent interventions

Interventions which combined two or more different components from other approaches (physical activity/exercise, social activities, health education, environment changes) were classified as multicomponent: 15 such studies, with a total of 13,350 participants, were reviewed. All 15 included some form of exercise or physical activity. In addition, ten interventions included social group activities, six studies organized health education, five studies provided psychological or motivation support, and in one study improvements were made to the built environment.

Analysis of the quantitative studies showed that when compared to the other intervention groups, multicomponent interventions had a greater positive effect on both social participation outcomes (81.8% of studies) and physical activity/fitness outcomes (80% of studies). The results of the qualitative studies support this finding.

## Discussion

This review has summarized evidence on the effects of community-based interventions which aimed to promote social participation and physical activity in older adults. It focused on trials conducted within the communities. To the best of our knowledge, this is the first review that focused on investigating the effectiveness, rather than efficacy, of intervention studies. While this approach led to the inclusion of some studies with lower scientific evidence, it also describes the interventions used in real-world conditions, in as much as possible. Based on this, we are able to form recommendations for communities (see below).

This review summarized results from various intervention study designs including pre- and post-test, retrospective, controlled trials and randomized controlled trials. A variety of intervention types were identified and allocated to the physical activity interventions, social activities that included physical activity, health education intervention, environmental interventions, and multicomponent interventions.

The majority of quantitative studies reported positive effects of interventions on social participation (68% of studies) and physical activity (67% of studies). All included qualitative studies reported that participants believed the interventions had had positive effects on their physical activity and social participation. It could be argued that this is due to the fact that quantitative measures might often be less sensitive to change. However, it could also be disputed that qualitative inquiry is more prone to socially desirable answers.

Our findings that interventions promoting social participation and physical activity can have positive effects on older adults are in line with findings of other reviews on similar topics (8, 17, 44). The classification of interventions into different types made it possible to identify approaches that more often reported statistically significant positive changes in physical activity and social participation of participants. Interventions that provided only physical activity/exercise activities often showed more positive effects on physical activity/fitness outcomes, rather than on social participation, which is in line with the findings from another review (45). The majority of interventions which focused on social activities had positive effects on social participation outcomes and nearly no effect on physical activity/fitness levels. The effect of health education interventions is rather unclear. It was impossible to make a comparison regarding environmental interventions, since only one such study with a small number of participants (ten older adults) has been identified. Further research needs to be done on these types of interventions in the future. In agreement with the review from Zubala et al. (8), we found that in quantitative studies on multicomponent interventions, more than 80% reported statistically significant effects on both social participation and physical activity/fitness outcomes – the highest result among all intervention types. This is in line with principles of ecological perspectives on health behavior change (46).

We observed a high variability in measures on physical activity and social participation outcomes across trials. This finding is in line with previous reviews on similar topics (17, 44) and this fact prevents comparisons between studies and interventions. This heterogeneity allowed us to summarize data only narratively and prevented a quantitative meta-analysis.

The methodological quality of the studies was also very heterogeneous, which has previously been noted in other reviews on similar topics (18, 44). The methodological quality of the reviewed studies was limited due to a lack of appropriate control conditions, consideration of cofounders and poor sample representatives (small number of participants, high loss to follow-up). There were many trials that reported on a small number of participants. In quantitative studies, randomization, confounding, and subject selection were often poorly described. In qualitative studies, authors often did not reflect on how the personal characteristics of researchers and their methods could have impacted the obtained data. Blinding of the participants was often not implemented as it is in general complicated for physical activity interventions. From the 23 studies where blinding of investigators was potentially possible, less than half (ten studies) used this opportunity and at least partly described the process. It is recommended that future studies find a way to blind at least investigators when it is possible.

More than 70% of participants in the reviewed studies were female and there seemed to be few interventions that particularly target males. Consequently, women seem to be overrepresented in the studies, even when considering that with 55% of the global population aged 65 years or over women comprise a majority of older persons worldwide (47). This might also be due to the fact that older women are much more likely to attend health prevention programs and community-based health screening programs than older men (48, 49). In a study from Sims-Gould et al. male participants reported that knowing that other men are also participating in a program is an important and attractive feature (50). The development and evaluation of programs which are more geared toward men or which are of more interest to a male audience, such as the “Men's Sheds” (51), can be suggested as a direction for future research.

The included studies did not specifically address minorities and socially disadvantaged groups or report data regarding potential health disparities (52). Thus, it remains unclear whether these interventions are suitable to prevent health inequalities or, on the other hand, if they might potentially widen the health gap between advantaged and disadvantaged groups of older people.

### Guidance to communities

Our main findings can be framed as recommendations to project officers working in communities to provide services to older people. Considering the findings of our review and the varied effectiveness of the different types of interventions to increase social participation and physical activity, certain (although preliminary) conclusions can be drawn:

- Health behavior and health education interventions that predominantly rely on lecturing and counseling do not work well. This type of interventions should only be conducted when there are special occasions that warrant this.
- Physical activity/exercise interventions increase physical activity, and social activities interventions increase social participation. Depending on the aim, one can choose which of the two to conduct accordingly.
- Physical activity/exercise interventions are more likely to increase social participation than social activity interventions are to increase physical activity. Due to the additional health benefits that come through physical activity/exercise interventions, these interventions might be a “best buy” for older people who are not too frail to participate in a physical activity program.
- Multicomponent interventions that combine social and physical activities had the highest rate of statistically positive results in comparison with other types of interventions. Services which are able to combine these different types of activities (e.g., health education, physical activity, and environmental measures) may be most beneficial.
- Specific efforts are required to reach minority or socially disadvantaged populations to prevent health inequalities and pursue health equity. Additionally, interventions specifically addressing the needs and interests of men are needed to attract this target group.

The decisive factors in choosing the type of intervention are undoubtedly the resources existing in the community and the characteristics of the population living in it. However, we recommend that communities take other factors into account that may generally contribute to or, conversely, hinder the implementation and promotion of such interventions. Organizers should consider in advance: the strategies for attracting participants to the program, the availability of the program venue, and the number and the necessary number of staff to conduct regular classes (50).

### Strengths and limitations

To the best of our knowledge, this is the first review focused on the effectiveness of interventions aiming to simultaneously promote physical activity and social participation among community-dwelling older people. The systematic search of relevant studies, with various study designs, was conducted in six electronic databases, and the methodological quality (risk of bias) was evaluated for all included studies. We see it as a strength of our study, that we were able to include 45 trials and draw some, although limited, conclusions regarding the effectiveness of different types of interventions.

We faced certain difficulties when conducting the review. From the outset, it was decided that an effectiveness review would need to be conducted, rather than an efficacy review (23). We were thus interested in trials that had been conducted in real-world settings, with free-living populations. This turned out to be difficult to determine for some studies, since the contexts within which trials were conducted and the recruitment of participants were sometimes ambiguously described.

Distinguishing between physical activity/exercise, social activities including physical activity, health education, and multicomponent interventions in the analysis was challenging, and for some interventions might have resulted in classifications that could be disputed. This was partially due to the fact that, like in other health behavior reviews, interventions were sometimes described only cursorily. This type of classification can also be regarded as being somewhat arbitrary. A dance class for example could, depending on one's perspective, be classified as an exercise intervention, a social activity, or a multicomponent intervention. We attempted, however, to describe the content of the intervention since it was our intention to provide project officers with some guidance on what to do. Additionally, most trials did not focus on minority or socially disadvantaged populations. This limits the generalizability of our findings.

This systematic review focused only on the effects of the interventions and did not provide information on the implementation process, such as how participants were recruited, drop-off rates, or protentional challenges. It could be recommended to focus on implementation effectiveness in further research.

## Conclusions

This systematic review sheds light on the effectiveness of community-based programs promoting physical activity and social participation for older people. Notwithstanding the large variability in study designs and measurements, overall, the findings from this review show that among the existing interventions there are some that can simultaneously increase physical activity and improve social participation among older people. The evidence also highlights multicomponent interventions' higher probability to show positive effects. The analyzed studies allowed for the formulation of some recommendations for communities who aim to promote physical activity and social participation in older adults. Further research on the effectiveness of interventions is recommended, especially in the field of rarely studied interventions, such as environmental interventions.

## Data availability statement

The original contributions presented in the study are included in the article/Supplementary material, further inquiries can be directed to the corresponding author.

## Author contributions

AT and VH conducted the literature search. AT, KA-O, and VH performed abstract/title screening, full-text selection, and data extraction. DM participated in the quality assessment. AT and KA-O analyzed data and summarized results. AT and KA-O with support from DM prepared a first draft of the manuscript. VH, AB, CC, CR, and AR critically revised the draft. AR supervised this work. All authors contributed to conceptualization of the study and approved the final manuscript.
