# Supplementary material for: Interventions simultaneously promoting social participation and physical activity in community living older adults: A systematic review
Source: Front Public Health. 2022 Dec 7;10:1048496. doi: 10.3389/fpubh.2022.1048496 (PMC9768837; doi:10.3389/fpubh.2022.1048496)
Supplement: Supplementary file 1 [file Table_1.DOCX]

Appendix 1. Data extraction table

| First author, year, country | Intervention description | Sample description | Measurement instruments related to social participation and PA/fitness | Effects on social participation (e.g., social support, loneliness, social contacts) | Effects on PA/ fitness (e.g., total PA, steps, timed up and go) |  |
| --- | --- | --- | --- | --- | --- | --- |
| PA/exercise intervention | | | | | | |
| Arnett,  2019,  USA | Fit and Fall Proof (FFP) group PA program to reduce falling risk by improving function and mobility. A 10-week program delivered 2-3 times per week. | N = 120 (no control group)  Age [years]: M = 76.1, range = 49-95  79.2% female | Functional fitness: the 8- foot Up and Go Test. At baseline, after first session (10 weeks), after second session (20 weeks).  Opened-ended survey collected from participants at the end of the first session (10 weeks) and the second session (20 weeks). | Based on answers to open ended questions, the most common self-reported benefit (37% of responses) was the opportunity for social engagement. | The results of the eight-foot TUG test showed significant improvement in functional fitness after 10 and 20 weeks compared to baseline (p < 0.000). | |
| Barbosa,  2018,  Brazil | 12-week aquatic training program (2 times/week, 1 hour/session) | ≥ 60 years old, without physical limitations which impede their ability to perform physical exercise.  N = 28,  Age [years]: M = 66.0, SD = 5.8,  89.3% female | Social participation: WHOQOL-Old questionnaire (subscale social participation)  At baseline and after 12 weeks. | No significant effect on social participation. | - |  |
| Barraagan,  2021,  USA | Community-based fitness program (CBFP) | 60-88 years old  N = 55 (no control group)  Age [years]: M = 72.0, SD = 7.2, 100% female | Focus groups  Time in the CBFP ranged from less than one year to 10 years (M = 3.64, SD = 2.65) | Participants attributed the CBFP to building social relationships. They found the environment to be welcoming, and enjoyed the predictable/ well-structured routine. This structure and purpose motivated them to attend regularly and helped them feel that they were productively contributing to the community.  CBFP also fostered a sense of belonging. There was a strength in shared experiences, which brought people together in an extraordinary way.  Additionally, participants mentioned that CBFP offers social benefits for passive and active participants. The group created a safe space where people felt comfortable regardless of their physical or social level. | - |  |
| Bidonde,  2009,  Canada | Fitness program 2 days per week for 60 minutes. | 65-84 years old, female, currently involved in the fitness program for at least 6 months, living alone in their own homes, independent in personal care, able to participate in interview, not currently involved in a community program for social isolation, and retired.  N = 9 (no control group)  Age [years]: M = 75  100% female | Semi-structured face-to-face interviews | Participants expressed gratitude for the opportunity to get out and be among people with similar life experiences in a group fitness environment. They  felt that their encountered and fostered a social support network through their involvement in the program, which allowed them to get out, socialize, and meet new people. | Participants mentioned improvements in strength, balance,flexibility, and bone health. |  |
| Brustio,  2018,  Italy | A dance training program that included different dance routines and was progressive in terms of motor complexity. Two 60-min sessions per week, 16 consecutive weeks for a total of 32 classes | ≥ 65 years old, retired, living independently, having a medical certificate of good health and able to walk without assistance.  N = 163 (no control group)  Age [years]: M = 70, SD = 4  75.5% female | Social engagement: LSNS-6  Mobility: TUG; TUG in a dual-task condition (TUGM);FSST  At baseline and after 16 weeks. | Significant improvement in LSNS-6 scores (p < 0.001). | Significant improvement in all mobility tests: TUG (p < 0.001), TUGM (p < 0.05) and FSS (p < 0.001). | |
| Carrapatosoa,  2017,  Portugal | 10- month walking program included individually reported functional fitness monitoring; monthly mail with educational materials and a schedule of the following months’ activities; three group walking/exercise sessions (40-50 min) per week; monthly activity “walking through the villages.; advertising panel with walking route information. | Retired, non-active in the previous year (engaging in at least two days a week for 20 minutes or more of MVPA)  N = 19 (no control group)  Age [years]: M = 67.4, SD = 2.5,  84.2% female  Interviews n = 12  83.3% female | Functional fitness: SFT  PA: IPAQ-SF  At baseline and after 10 months.  Semi-structured interviews after 10 months of participating in program | “Walking in a group” as well as “family” and “friends” were reported as being important social supports for the walking practice.  However, the participants also reported that social criticism was raised by some people in the city to the walking group, and the group decided to change the walking paths from the city area to more natural surroundings. | The scores of the functional fitness tests were significantly better after 10 months of the intervention, for all tests (p ≤ 0.02) apart from back stretching, seat, and reaching.  There was significant improvement in overall PA minutes per week (p ≤ 0.00) and walking minutes per week (p = 0.04).The interviewed participants (67%) reported improvements in their body weight, agility, strength, flexibility and aerobic endurance, which led to less fatigue when walking and climbing stairs. |  |
| Cedergren,  2007,  USA | Chair Volleyball Program - a competitive group PA. Games between teams registered in the league took place regularly from March to October. Additionally, each team practices weekly all year-round. | ≥ 55 years old  N = 222 (no control group)  79.7% female | Perceived social health benefits: 22-item survey instrument. | High ratings of perceived social health benefits related to social participation: 3.59 - 4.24 (out of 5 points).  Females and players who self-identified as very or extremely competitive rated these benefits higher. | - |  |
| Chan,  2017,  China (Hong Kong) | 3-month tai chi qigong program with the assistance of elderly neighborhood volunteers. Program included 60-minute exercise classes twice a week. Additionally, participants were encouraged to self-practice tai chi qigong for 30 minutes every day. The control group received usual care. | ≥ 60 years old; not engaged in any social activities  N = 48 (intervention group n = 24, control group n = 22)  Age [years]: M = 77.3, SD = 7.4,  76% female | Social network: LSNS-6  Loneliness: the De Jong Gieveld  loneliness scale  Social support: revised SSQ (6-item version).  At baseline (T0), post-intervention after three months (T1), and follow-up after six months (T2). | The tai chi qigong group had significantly greater improvements on the loneliness scale (p = 0.033) and the satisfaction component of the SSQ (p = 0.044) than the control group. | - | |
| Dionigi,  2007,  Australia | 12-week program of moderate-to-high intensity resistance training twice a week. | ≥ 65 years old  N = 10 (no control group)  Age [years]: range = 65-72  60% female | Interviews were conducted  1-week preintervention,  1 month after commencement, and 1 week after completion | Social interaction was associated with feelings of health and well-being. The participants enjoyed the opportunities for positive social interactions (including social support and social influences) with gym users, the other participants, and their assistant. | Already after 1 month of training many participants noticed improvements in their strength, endurance, balance, and coordination. |  |
| Ehlers,  2017,  USA | 24-week group-based exercise intervention that included 4 types of exercise groups: dance, strength/stretching/stability (SSS), walking, and walking plus (with nutrition supplements).  Each group attended three 1-hour exercise sessions per week. | 60–79 years old; able to read and speak English; right-handed; low-activity levels or inactive (i.e., 30 mins of moderate PA fewer than 2 days/week in the past 6 months); not involved in another PA program; without cognitive impairment.  N = 247 (Dance n = 69, Strength/Stretching/Stability n = 70, Walk n = 54, and Walk Plus n = 54)  Age [years]: M = 65.4, SD = 4.6, 68.4% female | Loneliness: UCLA Loneliness Scale.  Social support: SPS.  At baseline and after 6 months. | Perceived social support significantly increased (p < 0.01) and perceived loneliness significantly decreased (p < 0.01) after 6 months of the intervention. Changes in social support and perceived loneliness were similar across the intervention conditions. | - |  |
| Figureira,  2012,  Brazil | Governmental 12-week health program focused on PA. 50-minute varied PA sessions (walking, hydrogymnastics, strengthening, and stretching) twice a week.  The control group was advised not to perform any PA during the 12-week intervention period. | ≥ 60 years old, suitable for physical activities according to the program’s multidisciplinary team expert opinion.  Control group n = 35 (Age [years]: M = 69.8, SD = 8.1, 62.5% female)  Experimental group n = 35 (Age [years]: M = 68.7, SD = 5.9, 74.3% female) | Social participation: WHOQOL-Old questionnaire (subscale social participation)  At baseline and after 12 weeks. | The experimental group showed significant best results on the post-test on social participation (p = 0.013). | - |  |
| Frei,  2019,  Switzerland | The 6-month CAPACITY intervention aimed at enabling and encouraging participants to organize structured walking groups in their neighborhoods. Participants received mapped walking trails and a smartphone to count steps and communicate with each other. Additionally, a kick-off event and informal group get-togethers were organized. | ≥ 60 years old, German speaking, not among the  highest 25th percentile in the 1-min sit-to-stand exercise capacity test  N = 29 (no control group)  Age [years]: M = 75.4, SD = 7.8  75.9% female | Social support: short version of the Social Support Questionnaire (F-SozU)  PA: daily steps and average number of minutes spent in MVPA, triaxial accelerometer (ActiGraph wGT3X-BT, Pensacola, FL, USA).  Exercise capacity: number of repetitions in 1-min sit-to-stand test  Interviews used to qualitatively assess the intervention.  At baseline and 6 months post intervention | No significant changes in perceived social support.  In follow-up interviews, participants reported that they liked the social aspects best (without mentioning walking). | Number of minutes in MVPA per day significantly improved from baseline to follow-up with a median of  7.9 min (IQR = - 7.0 + 25.6; p = 0.046)  No significant improvements in daily steps and 1-min sit-to-stand test.  Subjectively, 10 persons estimated that they walked more, 9 somewhat more, 4 a similar amount, and 2 estimated walking less than before the onset of the intervention. |  |
| Gomeñuka,  2019,  Brazil | Nordic walking and free walking group. Duration: 8 weeks with three weekly sessions (24 sessions in total). | 60-80 years old; untrained; non- smoking; not currently experiencing chronic pain, migraines, or nausea in day-to-day life; without a history of labyrinthitis, or any other health condition which could prevent or limit their ability to complete the sessions or assessments.  N = 33 (nordic walking: n = 16, age [M ± SD]= 64.6 ± 4.1 years; free walking: n = 17, age [M ±SD] = 68.6 ± 3.9 years)  72.7% female | Social participation: WHOQOL-Old questionnaire (subscale Social participation)  Static balance: test on a force platform; self-selected walking speed: treadmill test; dynamic stability: gait variability assessed during treadmill walking; closeness of self-selected walking speed to optimal walking speed: locomotor rehabilitation index  At baseline and after 8 weeks. | Significant improvement in social participation (p < 0.001) in both groups. | Improvements in the self-selected walking speed (p = 0.011), locomotor rehabilitation index (p = 0.013), static balance (p < 0.05) and dynamic variability (p < 0.05) in both training groups. |  |
| Kohut,  2006,  USA | Cardiovascular (CARDIO) exercise or strength/flexibility/balance (FLEX) exercise intervention. Sessions held 3 times per week during 10 months. | ≥ 64 years old, participation in aerobic exercise ≤ 3 times per week at 40% or less of heart rate reserve or aerobic fitness level below 75^th^ percentile of their age and sex  N = 87 (CARDIO group n = 40, FLEX group n = 47).  Mean age FLEX = 70.3 ± 4.6 years.  Age [years] CARDIO: M = 69.8, SD = 5.5,  65% female | Social support: SPS  Fitness level: SFT, maximal exercise treadmill test.  At baseline and after 10 months. | No significant time effect for the SPS, only a trend in increasing perceived social support (p = 0.07). | Both groups improved cardiorespiratory fitness (greater improvement in CARDIO group) (p < 0.001), leg strength (p = 0.028), upper body/arm strength (p < .001), shoulder flexibility (p = 0.04). |  |
| Komatsu,  2017,  Japan | The Fujisawa +10 exercise program included low intensive exercise sessions 1-3 times per week.  The older adults were also taught how to exercise and were given a CD, DVD, and a manual to support them in exercising together without an instructor. | ≥ 60 years old, who regularly participated in the group exercises, and lived independently without any elderly care assistance.  N = 26 (no control group),  Age [years]: M= 74.7, range: 66–86,  57.7% female | Focus groups | By participating in the group exercise regularly, the participants felt that they were socially connected and were members of a community.  The participants expanded their communication beyond the group exercise. They planned social activities with their peers, including excursions and eating out together.  With increased social activities, the older adults began to actively connect with each other. | Many of the participants perceived regular group exercise as good for their functional health. |  |
| Liu,  2014,  China (Hong Kong) | Taichi with and without a cognitive-behavioral intervention (CBI) to reduce fear of falling.  All participants received the 8-week tai chi coaching group sessions, with a duration of 60 minutes each.  In addition to the tai chi training, 8 weekly sessions of 60– 90 min CBI were implemented for participants of the tai chi plus CBI group. | ≥ 65 years old, fall within the previous 12 months, fear of falling, physical condition is stable enough to perform daily tai chi practices (e.g., they can stand on one leg for at least 5 s), able to communicate in Cantonese.  N = 122 (tai chi group n = 58, tai chi plus CBI n = 64).  Age [years]: M = 74.5, SD = 7.3  87% female. | Social activity participation: 5 social engagement related questions.  Mobility (balance and gait): Tinetti’s balance test and Tinetti’s gait test.  PA: average hours spent on exercise.  At baseline, after 8 weeks, and after 16 weeks. | No significant changes in social participation. | No significant improvement in balance, gait and PA level. |  |
| Maki,  2012,  Japan | The Takasaki Project /intervention encouraged participants to acquire a walking habit by gradually increasing their walking steps in a group setting. The 90-minute intervention program was conducted once a week for 12 weeks and consisted of a 30-minute exercise period and 60-minute group work with five to eight people. | 65-80 years old, at high risk of mental decline.  N = 150 (intervention group n = 75,  control group n = 75).  Age [years]: M = 72.0, SD = 4.0,  70.7% female | Social network: LSNS (abbreviated version)  Motor function: grip force, balance time on one foot, TUG, and maximum walking speed for 5 meters.  PA: average steps per day measured with the pedometer EX-500 (Yamasa Tokei Co. Ltd.) (7-day measurement)  At baseline and after 12 weeks. | No significant differences between the treatment and control group in social network size. | The intervention group had a significantly greater increase in average number of steps (F(1,123 ) = 7.184, p = 0.008) and the TUG (p = 0.002) from the pre- to the postintervention period than the control group.  No significant differences between the treatment and control groups in grip force, balance time, or walking speed tests |  |
| McAuley,  2000,  USA | One group participated in an aerobic activity program (walking) three times per week starting from 10-15 minutes per session progressing to 40 minutes per session.  Another group participated in a stretching and toning program three times per week for one hour.  Duration of both programs was 6 months. | 60-75 years old, and sedentary (not having participated in regular exercise over the previous 6 months).  N = 174 (aerobic n = 85, toning n = 89)  Age [years]: M = 66.7, SD = 5.4,  71.8% female | Social support: SPS.  Loneliness: UCLA Loneliness Scale  Social support: After two weeks of the intervention and during the last week of the intervention (6 month).  Loneliness: Before the intervention, during the last week of the intervention (6 month), and 6 months after intervention (12 month). | Loneliness significantly decreased at the end of the intervention with a small but significant increase remaining after 12 months. | - |  |
| Streber,  2017,  Germany | The 12-week GESTALT-kompakt intervention is a multimodal PA program with 12 sessions. Sessions lasted 90 min each and were held once a week (60 min of an evidence-based multimodal PA program integrating social and cognitive activities + 30 min of a PA coaching program).  Three independent groups – one memory training and two  PA groups – served as control groups. All sessions were  group based, took place once a week, and lasted 45–60 min. There was no simultaneous performance of physical and cognitive tasks. | ≥ 60 years old, at risk for dementia  N = 87 (intervention group n = 57, control group n = 30).  Age [years]: M = 76.0, SD = 9.2,  78.2% female | Social activity: the “Social Activity Log” questionnaire.  PA (average steps per day): waist- mounted pedometer (Fitbit Zip) (7-day-measurement)  At the baseline (T0), after 12 weeks of intervention (T1) and 12 months after the beginning of the intervention (T2). | Small time effects after 3 months in the control group and after 12 months (T0-T2) in the intervention group.  No overall intervention effect on social activities after 3 and 12 months. | There was a significant increase in the average number of steps after 3 months (T1) in the intervention group, but not in the control group. The interaction effect was not significant (866.4 steps, p = 0.055). From T1 to T2, there was no significant interaction effect, but a significant decrease in steps was detected in the intervention group. There was no overall effect of the intervention on the PA (T0-T2). |  |
| Wang,  2010,  USA | Yoga intervention group.  Participants in socialization comparison group were shown movies.  Both groups met twice a week for one 60 min sessions for four consecutive weeks. | ≥ 60 years old  N = 18 (yoga group n = 8, socialization group n = 10)  Age [years]: M = 74.9, SD = 8.4,  88.9% female | Social isolation: UCLA Loneliness Scale.  Balance: the one-leg stand test  Lower body strength: the sit-to-stand test.  Flexibility: the sit-and-reach test.  At baseline and after 4 weeks of intervention. | No significant difference between pre and post measures in the yoga group, significant decrease in loneliness in the socialization group (p = 0.025). | No significant difference between pre and post measures. |  |
| Wickman,  2017,  Denmark | Intervention group played floorball.  Control group played petanque (little PA or contact).  Both groups met for one hour twice a week for 12 weeks. | ≥ 60 years old and untrained (had not been involved in any type of regular (< 2 weekly sessions) intense physical training for at least 10 years)), men  N = 37 (floorball n = 22, petanque n = 15),  Age [years]: M = 69.8, SD = 3.7 | Semi-structured interview after 12-week intervention. | The interviewees mentioned the following factors as contributing to a high degree of social connectedness: the group only consisting of men age 65+ (common background), an inclusive environment where everyone had similar skill levels, and the fun they had while participating. Th men in the floorball group experienced a high degree of solidarity and group cohesion which seemed to have increased their social capital during the intervention. | Most of the interviewees named the aim of improving their fitness as their main motivation to begin floorball training. The participants were able to notice physical improvements in their everyday lives as a result of the training. |  |
| Social activities that included PA component | | | | | | |
| Austin,  2006,  USA | Community gardening. Each participant was provided space, plant containers, and plants (individually selected). | N = 6 (no control group)  Age [years]: M = 68.2, SD = 8.0,  50% female | Social support and social activities subscales of the Dartmouth COOP Functional Health Assessment Charts.  Physical fitness: 6MWT.  At baseline and after 8 weeks of the intervention. | The improvement in social support scores was not significant. Significant improvements in social activities subscale (p = 0.046). | No significant changes |  |
| Boyes,  2013,  New Zealand | The Third Age Adventures program provides opportunities for older individuals to participate in outdoor adventure activities. A variety of different trips (activity types and difficulty levels) are available to meet individual needs: from half-day walks and bike rides to multi-day backpacking trips. | For interviews n = 6,  63-80 years old,  67% female.  For survey N = 80 (no control group),  Age [years]: M = 67, range = 54-83,  63% female | Semi-structured interviews and surveys to explore the physical, social, and psychological benefits of the program. | Participants mentioned enjoying the social activities and discussions, and the chance to meet new people and integrate into the community.  This is reflected in the survey results, where participants rated their social experiences highly (total score 6.15 ± 0.99 out of 7).  The Third Age group was the strongest source of friendship networks (51% had many Third Age friendships) compared to family (41% had many connections) and other friendship networks (26%). | Participants mentioned during the interviews that they experienced health improvements since participating in program.  The following statements in the survey received the highest ratings: participants became more physically active, improved their physical fitness, have better endurance and higher energy levels.  33% of participants reported a link between engagement in the program and an improvement in blood pressure and blood lipid levels. |  |
| Da Silva,  2016,  Brazil | Dance evenings held at a community center once a week. | N = 12 (no control group)  Age [years]: M = 66.2, SD = 4.3,  66.7% female | Qualitative interviews. | Participants reported that dance helped them broaden and strengthen social ties and relationships. | Participants reported that dancing is a PA that trains the limbs (arms and legs), and helps reduce muscle and bone pain. |  |
| Gagliardi,  2018,  Italy | Social farming. The program took place over a minimum of 50 days. Agricultural activities included raised bed gardening, harvesting olives, pruning, breeding courtyard animals, crafting, food education, cooking, and physical exercise sessions. Intergenerational events were organized on six farms every week for 1 year. | ≥ 65 years old  N = 73 (no control group)  Age [years]: M = 72.9, SD = 8.6,  63% female | Social relationships: the frequency of contact with personal networks in the last 4 weeks.  PA: a question from the Minimum Data Set Home Care Assessment assessing hours of exercise performed in the previous 3 days  At baseline and after the year‐long farm program. | The participants’ contact with friends (p = 0.001) or relatives (p = 0.009) at least once a week significantly increased. The number of participants practicing home gardening increased from 24.3% to 50%. | No significant changes in PA level. |  |
| Gagliardi,  2020,  Italy | Program of environmental volunteering and social activities in city parks for older people. The program offered older participants the opportunity to experience outdoor activities, carried out in two city parks, through sustainable gardening practices. The activities took place twice a week. | N = 19 (no control group)  Age [years]: M = 75.7, SD = 5.1,  42.1% female | Social participation: LSNS (7-item version).  PA: PASE.  Experienced outcomes: Qualitative interviews.  At baseline and after participation in the program | The LSNS did not show significant variations, except for a significant increase in the frequency of interaction with relatives (p = 0.014).  Perceived effects based on qualitative interviews: social support including positive group and family interactions. | Significant increase in outdoor gardening (p = 0.021), caring for others (p = 0.042), aid/voluntary work over the past 7 days (p = 0.009), amount of PA required by job/volunteering (p < 0.001), and overall PASE scores (p = 0.013).Perceived effects based on the qualitative interviews: overcoming laziness, being more active. |  |
| Johnson,  2018,  USA | 44-week choir program “The Community of Voices”.  Participants attended 90 min choir sessions weekly. Each session included activities targeting three hypothesized pathways by which a choir could promote health and well-being: cognitive, physical, and psychosocial engagement.  Control group: not reported | ≥ 60 years old, with sufficient visual and hearing acuity; fluent in English or Spanish; without cognitive impairment or dementia  N = 390 (intervention group n = 208,  control group n = 182).  Age [years]: M = 71.3, SD = 7.2,  76.4% female | Loneliness: NIH Toolbox.  Lower body strength: SPPB (chair stand test)  Balance and gait speed: NIH Toolbox.  At baseline and after 6 months | Loneliness significantly decreased in the intervention group whereas the control group did not see any significant changes in loneliness. (significant group-by-time interaction effects (p = 0.02) | No significant improvement in chair stands, balance, or gait speed. |  |
| Health behavior intervention/Health education (lectures, counselling) | | | | | | |
| Franke,  2021,  Canada | Health promotion program Choose to Move (CTM),  Lasted for 3-months and included:  1) a 60-min one-on-one consultation with Activity Coaches to set goals and create a personalized PA Action Plan.  2) four 60-min Motivational Group Meetings (1x in months 1–2; 2x in month 3) to help participants socially connect with each other (max 12/group) and with their Activity Coaches. Group sessions included presentations and group discussions on lifestyle topics that supported the development and maintenance of a PA Action Plan  3) Activity Coaches called participants regularly by phone (15 min/call on average) to monitor progress, address challenges and modify the Action Plan as needed (3x in month 1; 2x in months 2 and 3; 1x in months 4–6). | ≥ 60 years, English speaking, and physically inactive (self-reported < 150min/week of PA)  N = 452 (no control group),  77% female  Sample divided in two groups:  Not lonely n = 191.  Lonely n = 261.  For qualitative part at baseline n = 43, 3 months n = 38, 6 months n = 19. | Loneliness: three item questionnaires (LQ-3), questions asked were, “how much of the time do you feel (i) you lack companionship; (ii) left out; (iii) isolated from others”  PA: self-reported PA (1 item: number of days/week ≥ 30 min PA in the past week, and capacity for mobility as no/any difficulty walking 400 m or climbing one flight of stairs (1 item))  Qualitative survey to evaluate how the program influenced social connections.  At baseline, 3 (mid-intervention) and 6 (post-intervention) months | In participants who identified as lonely at baseline, loneliness decreased significantly from 0 to 3 months and lower loneliness scores were maintained at 6 months (p < 0.001) There was no change in loneliness from 0 to 3 months in the ‘not lonely’ group. However, loneliness increased significantly in this group at 6 months compared to baseline (p = 0.006).  In a qualitative survey, participants mentioned motivational Group meetings having the following factors which promote social connectedness /reduce loneliness:  - Activity Coaches characteristics/personality traits and approaches  - Opportunities to share information/experiences and learn from others  - Interaction with others who share similar/familiar experiences  - Increased opportunity for meaningful interactions | PA increased significantly during the active intervention phase (baseline to 3 months) in both lonely and not lonely participants (p < 0.001). PA decreased significantly from 3 to 6 months in lonely participants. However, PA at 6 months remained significantly above baseline levels in both groups (p < 0.001). |  |
| McKay,  2018,  Canada | The health promotion program Choose to Move (CTM), is a  3-month intervention which included:  1) a 60-min one-on-one consultation with Activity Coaches  2) Four 60-min Motivational Group Meetings to socially connect participants with other participants and their Activity Coaches. Group sessions included presentations and group discussions on lifestyle topics that support developing and sustaining a PA Action Plan.  3) Activity Coaches regularly called participants on the phone (15 min calls on average) to monitor progress, address challenges, and modify Action Plans as needed (3x in month 1; 2x in months 2 and 3; 1x in months 4–6). | ≥ 60 years old, English speaking, and physically inactive (self-reported < 150min of PA per week)  N = 458 (no control group),  77% female  Results reported by age groups:  Younger 60-74 years n = 323,  Older ≥ 75 years n = 135 | Social connectedness: 3-item questionnaire -  “How often do you (i) get together with friends, neighbors, or relatives, and do things like go out together or visit in each other’s homes?; (ii) talk on the telephone or exchange emails with friends, neighbors or relatives? and; (iii) attend meetings or programs of groups, clubs or organizations that you belong to?”  Loneliness: 3-item questionnaire (LQ-3):  “how much of the time do you feel (i) you lack companionship; (ii) left out; (iii) isolated from others”  PA: self-reported PA (1 item: number of days with ≥ 30 min PA in the past week)  Capacity for mobility as no/any difficulty walking 400 m or climbing one flight of stairs (1 item))  At baseline, 3 months (mid-intervention) and 6 months (post-intervention) | There was no change in social exclusion over time in older participants.  Social exclusion indicators declined significantly in the younger group from baseline 3 (p < 0.001) and 6 months (p = 0.02).  Loneliness decreased significantly during the active intervention phase (baseline to 3months) in both younger (p < 0.001) and older (p < 0.001) participants. Loneliness remained lower at 6 months compared to baseline in both younger (p < 0.001) and older (p = 0.01) participants. | PA increased significantly during the active intervention phase (baseline-3 months) in both age groups (p < 0.001). The increase was only sustained at 6 months in younger participants (p < 0.001).  Mobility limitation decreased significantly in the younger group both at 3 months (p < 0.001) and 6 months (p = 0.002) compared to baseline. In older participants, mobility limitations were significantly decreased at 3 months (p = 0.02), but not at 6 months (p = 0.07) |  |
| Mendoza-Ruvalcaba,  2016,  Mexico | Vital Aging is a program to promote active aging through teaching basic knowledge about aging, promoting healthy behavioral lifestyles (physical exercise, nutrition), training strategies for optimizing  cognitive functioning and compensating potential cognitive declines, optimizing positive affect, emotion, and control, and promoting social relationships and social engagement.  In Vital Aging face-to-face (VA-FF) version of program participants attend 2/3-hour group sessions, held twice a week during  10 weeks.  In Vital Aging combined (VA-C) version of program 11 of 18 sessions were provide in multimedia format, other 7 in face-to-face format.  Control group participants remained on a wait list and participated in usual activities. | ≥ 60years  N = 76 (VA-FF n = 35, VA-C n = 15, control n = 26),  Age [years]: M = 65.7; SD = 6.5,  100% female. | Social interactions: Frequency of social interactions with family, friends, and neighbors. Response options range from 1 = less than once a month to 5 = several times a week.  Satisfaction with social relationships measures the degree to which one is satisfied with their relationships with family, friends, and neighbors (1 = not satisfied to 5 = very; satisfied).  PA: The level and frequency of PA in the past month (answers range from 1 = sedentary to 5 = intense exercise, 3 times a week). | No significant increase on the frequency of interactions with family, friends, and neighbors in all groups.  Participants of the face-to-face group reported higher satisfaction with their relationships after the program. | No effects in PA in all groups. |  |
| Multicomponent | | | | | | |
| Ahn,  2014,  South Korea | Integrated Health Management Program. The intervention included exercise, health education, and social activities, and was performed 2.5 hrs. per week for 12 weeks | ≥ 60 years old, females, not exercising regularly, not having participated in another health program in the past month, without serious health concerns, were not undergoing diagnosis or receiving treatment for depression or dementia, and having normal cognitive functions.  N = 33 (no control group)  Age [years]: M = 70.0, SD = 4.9 | Social support: MSPSS  Physical fitness: skeletal muscle mass using the InBody 330, arm grip strength, chair stand, chair sit-and-reach, and the straight walking test.  At baseline and after 12 weeks of intervention | Social support scores significantly increased (p < 0.001). | Skeletal muscle mass significantly increased (p = 0.002). Chair stand (p = 0.023) and straight walking test (p < 0.001) were significantly improved.  There was no change in arm grip strength (p = 0.626) and in the chair sit-and- reach test (p = 0.386) |  |
| Cardenas,  2009,  USA | North Carolina Senior Games (NCSG) is a community-based recreation program that aimed at encouraging older adults to be active year-round in sports and fitness programs as well as creative arts. | ≥ 55 years old  N = 444 (no control group),  Age [years]: M = 70, range = 55-96  52% female | The Behavioral Risk Factor Surveillance System (BRFSS),  Questionnaire that examined the nature of the experience included whether training or preparing for NCSG was a part of weekly activities (yes/no) and perceptions of whether NCSG provides motivation to be more physically or socially active (yes/no) | 66% participants reported that participating in NCSG motivates them to be more socially active. | The data indicated that 60% of the 55–64-year-old NCSG respondents were moderately active compared to 40% of that age group in the 2005 BRFSS. For individuals 65 years and older, almost twice as many NCSG older adults (64%) said they were moderately active- compared to BRFSS statistics which indicated only 34% of individuals in that age group.  61% of the respondents said that participating in NCSG motivates them to be more physically active. |  |
| Hopman-Rock,  2002,  Netherlands | The Aging Well and Healthy (AWH) program consists of health education by peers and low-intensity exercise. It consists of six sessions- each including 1 hour of health education and 1 hour of exercises. | ≥ 55 years old  Intervention group n = 390,  Age [years]: M = 72.3, SD = 6.0,  82% female.  Control group n = 156,  Age [years]: M = 73.6, SD = 6.0,  89% female | Loneliness: Question, “I feel lonely” with possible answers 1-5 (5 = not lonely at all).  PA: Questionnaire designed for use with older adults (Voorrips et al., 1991).  Pre-test, post-test and follow-up (4-6 month after the AWH program) | The mean scores for loneliness tended to improve at post-test measurement (p = 0.08) and significantly improved at follow-up (p = 0.00) | PA scores significantly increased at post-test measurement (p = 0.00).  25% of participants joined exercise groups after the program ended, and 28% intended to do so.  4-6 month after the program, 60% of participants reported still doing exercises regularly at home. |  |
| Huang,  2011  Taiwan | Tai chi combined cognitive- behavioral (CB) intervention compared with only CB and a control group who received no extra care.  CB intervention consisted of 8-weekly group sessions, lasting 60–90 minutes.  Tai chi group received the same CB intervention and additionally tai chi 1-hour lessons 5 times a week, participants had to complete three sessions per week for 8 weeks. | ≥ 60 years old, mentally intact, residing in the community, and able to communicate in Mandarin or Taiwanese.  N = 186 (tai chi + CB n = 62, CB n = 62, control n = 62)  60-64 years n = 35,  ≥ 65 years n = 151  58.6% female | Social support behaviors and satisfaction: ISSB (Chinese version)  Mobility (gait and balance): the Tinetti Mobility Scale.  At baseline, 2 months, and 5 months. | tai chi + CB group had significantly higher social support satisfaction (p < 0.001) than the CB alone and control groups at 5 months. | tai chi + CB group had significantly higher mean mobility scores (giant and balance) (p < 0.001) than the CB alone and control groups at 5 months. |  |
| Hwang,  2019,  Canada | Walk ‘n’ Talk for Your Life (WTL) 12-week program. Sessions occurred twice a week and consisted of a 45-minute fitness program designed to reduce falls and fall-related injuries, a 30-minute pedometer-based group walk, followed by a 60-minute interactive health education session and 20-minute open socialization | Having completed the 12-week WTL program, not self-reporting a loss of hearing, and being available during scheduled interview times.  N = 16,  Age [years]: M = 76.6, range: 65-88,  94% female | Semi-structured interviews were used to provide insight into participants’ experiences of loneliness, social isolation, and the WTL program. | Participants talked about improvements in their social networks and sense of belonging as effects of the program. Some described improved social connections and social supports. | Participants attributed the program to self-improvements in extraversion, learning new things about their neighborhood, and improving their physical wellbeing. Some also noticed an improvement in motivation, such as increased determination to reach physical goals, or to get out of the house. |  |
| Kamegaya,  2013,  Japan | 12-week physical and leisure activity program. The intervention group attended a weekly 2-h program that included 45-minute PA sessions (stretching, strengthening and aerobic exercises) and leisure activities, such as cooking, handcrafts, and competitive games.  Participants in the control group did not attend a program during this period. | ≥ 65 years old  N = 52 (no control group)  Age [years]: M = 74.9, SD = 5.9,  90.4% female | Social support: LSNS-R  Physical fitness: grip strength test, TUG, 5-m maximum walking times test, and functional reach test | No significant differences were observed in social support. | None of the items on the physical function tests showed significant changes in the intervention group. |  |
| Kim,  2020,  South Korea | Integrated Healthcare Program for the Rural Older Adults. 12 2-hr sessions once a week, each including activities to increase physical function, ego integration, and role self-efficacy, and to decrease loneliness (lectures, exercises, group activities) | ≥ 65 years old, being a rural community-resident, being able to communicate, and to understand the study purpose and provide written consent to voluntarily participate.  Intervention group n = 39,  Age [years]: M = 74.7, SD =  5.3,  89.7% female.  Control group n = 38, Age: M = 76.6, SD = 5.5 years,  76.3% female | Loneliness: UCLA Loneliness Scale.  Physical functions: left and right upper extremity strength was measured using a dynamometer. Static balance was measured per second standing on one foot with open eyes.  Dynamic balance: 8-foot Up-and-Go Test.  At baseline and after 12 weeks of intervention | There was a meaningful difference in loneliness (p = 0.003) of the two groups, with the experimental group decreasing from 30.46 ± 3.27 before the program to 28.69 ± 3.13 after, while the control group increased from 29.24 ± 2.57 before the program to 29.79 ± 2.80. | Compared with the control group, the intervention group demonstrated a significant increase of strength in both upper extremities (right p = 0.008; left p = 0.047), static balance (p = 0.017), dynamic balance (p < 0.001). |  |
| McMahon,  2016,  USA | The 8-week fall-reducing intervention Ready-Steady combined two components: (1) motivational (motivational support, social network support, empowering education), and (2) fall-reducing PAs (PAs; guidance to practice leg-strengthening, balance, and flexibility activities and walking).  It included one 90-minute session a week, that took place in small groups.  Control participants received information about health and wellness topics: falls; eye and foot health; home, environmental, and medication safety; sleep; hearing; and hydration and nutrition. | ≥ 74 years old, rural, PA levels below recommended guidelines for aerobic and muscle-strengthening activities.  N = 30 (n = 16 for intervention group, n = 14 for Attention Control group)  Age [years]: M = 83.6, SD =4.7  93.3% female | Social support: Social Support for Exercise Questionnaire (SSES)  PA: Total weekly duration measured with the Community Health Activities Model Program for Seniors questionnaire (CHAMPS), triaxial accelerometer (mHealth app).  Balance, strength, gait velocity: SPPB.  At baseline (1 week before intervention) and 1 week after intervention. | There was a significant increase among the  intervention group of reported support from friends to exercise (p = 0.002). | The intervention group improved significantly more than the attention-control group in CHAMPS (p = 0.002), accelerometer (p = 0.02), and SPPB balance and lower extremity strength (p = 0.001). | |
| McNamara,  2016,  Australia | Healthy ageing intervention which included age-appropriate forms of exercise (e.g., ‘heart moves’, belly dancing, line dancing, Feldenkrais movement) and a social component (e.g., morning tea and social interaction).  The program lasted eight weeks, with a two-hour session every week. | ≥ 55 years old with an ongoing functional disability which impacts their activities of daily living  N = 21 (no control group),  71% female | Interview.  Physical functioning: SF-36 (physical functioning domain)  At baseline, after intervention, follow-up telephone interview | According to the interview results, the program provided participants with an opportunity to meet new people and ‘get out of the house’. | The follow-up telephone interviews revealed that more than half of the participants still practiced the exercises they had learned from the program. Others reported not needing to visit the physiotherapist as often, as they did more exercises at home. |  |
| Merchant,  2021,  Singapore | The Healthy Ageing Promotion Program for You (HAPPY) is a dual-task exercise program incorporating physical, cognitive, and social activities. The exercises are conducted for 60 minutes once or twice weekly. | ≥ 60 years old, prefrail or frail, with ambulant or cognitive impairment.  N = 197 (no control group)  75% female | Social network: LSNS-6.  Balance, gait speed, and leg strength: SPPB.  At baseline and after 3 months. | Significant improvement in LSNS-6 scores with reduction of social isolation by 10% at 3 months. | Significant improvement in SPPB scores. Significant improvement in the SPPB domains of balance and chair-stand.  No significant improvements in the SPPB domain gait speed. | |
| Ren,  2021,  China | Group Reminiscence Therapy in Combination with  Physical Exercise. Duration of 8 weeks.  Intervention included:  1) Community health education, 4 lectures every two weeks.  2) Psychological intervention: “reminiscence club” activity was organized once a week, 50-60 minutes per session.  3) Taijiquan exercise three times a week, 45 minutes each session.  Control group participated solely in the health education lectures. | ≥ 60 years old; with normal perception and language communication skills; were able to participate in physical exercise.  N = 121 (intervention group n = 60, control group n = 61). | Loneliness: ULS Loneliness Scale.  At baseline and after 8 weeks of intervention. | Loneliness significantly decreased in both groups (P < 0.001).  The intervention group had significantly lower scores in loneliness scale (p = 0.047) and the decrease in the loneliness score after the intervention was statistically significantly higher in the experimental group than in the control group (p = 0.001). | - |  |
| Seino,  2021,  Japan | “Ota Genki Senior Project” 2-year community-wide participatory intervention (CWI). The intervention aimed to 1) establish resident-oriented activities related to frailty prevention (ie, physical exercise, nutrition, and/or social participation), and 2) improve the community environment (ie, communicating the importance of exercise activities, dietary variety, and active social participation by cooperating organizations and implementing these as part of existing efforts). | ≥ 65 years old.  Intervention group n = 6009,  Age: M = 74.3, SD = 5.4 years,  51.7% female.  Control group n = 5692, Age [years]: M = 74.3, SD = 5.5,  51.4% female | Social participation: self-administered questionnaire to assess  going outdoors ≥ 1 time/day, social isolation, and cognitive and structural social capital  PA and physical function: engaging in any exercise ≥ 1 time/week and walking ≥ 150 min/week; the Motor Fitness Scale.  At baseline and after 2 years. | No significant differences in social participation between intervention and control groups.  Subgroup analyses showed increasing in going outdoors ≥ 1 time/day (2.1 percentage points [0.1–4.1]) in district C were observed, as compared with the control group. | No significant differences in PA and physical functioning between intervention and control groups.  Subgroup analyses showed a benefit for ≥ 150 min/week of walking (3.9 PP [1.9–5.8]) in district A, ≥ 1 time/week (2.1 PP [0.1–4.0]) for exercise, and a difference of .10 points in the Motor Fitness Scale (0.10 points [0.01–0.20]) in district C, in comparison to control group. |  |
| Shvedko,  2020,  UK | Physical Activity Intervention for Loneliness (PAIL) is a 12-week intervention consisting of group walks and health educational/social interaction workshops performed once weekly for a duration of up to 90 min per session.  The control group were individuals on a wait list. | ≥ 60 years old, sedentary (less than 20 min of MVPA a week), at risk of loneliness, physically mobile, English speaking and able to complete handwritten questionnaires.  N = 25 (intervention group n = 12, control group n = 13)  Age [years]: M= 68.5, SD = 8.1  56% female  Focus groups n = 5 | Focus groups at the middle of intervention (4-5 week) and at the end- 12 weeks. | Focus group participants mentioned that participation in the program was a chance to meet new people and get access to local community groups, walking was believed to have promoted bonding between participants and to have improved their aspirations for friendship-based relationships. | Focus group participants felt that participation in the intervention helped them to become more physically active, which was their initial aim. |  |
| Yamamoto,  2020,  Japan | 5-week combined exercise and education program. 5 sessions once a week which included 60 minutes of exercise and 20 minutes of an educational program about a health-related topic. | ≥ 65 years old  N = 31(no control group)  Age [years]: M= 70.9, SD = 4.5  56% female | Social network: LSNS-6.  Physical performance: 30-second chair stand test, TUG, the maximal isometric torque test.  At baseline, after 5 weeks of intervention, and 1 month after intervention. | Social engagement was significantly higher at the end of the intervention than at baseline (p = 0.02), but this improvement was not maintained in the follow-up assessment. | There was a significant improvement in the 30-second chair stand test (p < 0.001) and TUG (p < 0.001) at pre and post measurements. |  |
| Yeom,  2014,  USA | Motivational Physical Activity Intervention (MPAI).  1-hour sessions held twice a week for 12 weeks.  MPAI critical inputs included (a) PA training, including 10-min warm up using flexibility exercises, 10-min balance training, and 20-min moderate- intensity walking; (b) social support operationalized through group process, goal setting, and interaction; (c) empowering education focused on creating social contextual resources; and (d) motivational support for enhancing motivational appraisal and skills to initiate and sustain regular PA.  The Attention Control group received biweekly newsletters over the same time period, focusing on older adult health and general issues, such as healthy  nutrition, stress management, oral health, home safety, language issues, and interpersonal relationships. | ≥ 60 years old, Community-dwelling Korean Americans, long-term residents, currently sedentary, able to communicate in Korean or English, having intact cognition, and at low risk for participation in moderate-intensity physical activity.  N = 64 (n = 33 for MPAI group, n = 31 for Attention Control group)  Age [years]: M = 71.0, SD = 7.4  76.6% female | Social support: the Social Support and Exercise Survey.    PA: single item, “Do you currently do any type of regular physical activity for a minimum of 30 min 3 times a week?”.    Walking endurance: 6MWT  Balance, flexibility, and gait velocity: SPPB.  At baseline and after 13 weeks | Scores of MPAI participants increased significantly compared with Attention Control participants in social support from family (p < 0.001) and friends (p < 0.001). | Following the intervention, MPAI participants were more likely to engage in regular PA compared with the control group (p < 0.001).  MPAI participants had significant improvement in 6MWT (p < 0.001) and in flexibility (p < 0.001) compared to the control group. There were no significant differences between the intervention and control group in balance or gait velocity scores (SPPB). | |
| Environmental intervention | | | | | | |
| Schmidt,  2020,  Denmark | “Move the Neighborhood” program for increasing the use of neighborhood open spaces (NOS) to promote active living through social interaction and PA. Based on the participatory research approach. Local older adults with landscape architects jointly explored and developed ideas on how to improve NOS. The renovations were made based on the results of this collaboration. | For qualitative interviews N = 10  Age [years]: M = 77  74.2% female | The System for Observing Play and Recreation in Communities (SOPARC) to collect information on social interaction (two or more persons talking, walking running, biking, sitting together) and activity level (sedentary, walking [light/moderate], vigorous).  Measured before and after NOS renovation.  Qualitative interviews after intervention. | The percentage of older adults engaged in social interaction increased from 31.1% at baseline to 53.8% at follow-up.  The highest increase in social interaction was observed in the NOS with the renovated benches.  Interviewees stated that having someone to talk to or sit with within the NOS was an important factor, as it was a reason for them to go out and use their NOS.  Interviewed individuals stated the importance of having an NOS close to their home which attracts residents and creates a pleasant environment- where people don't have to organize an event to socially interact, but rather can meet and greet people there on a casual, spontaneous basis. | The number of people using NOS increased. At baseline, 209 older adults were observed (18.2% from all visitors), whereas 329 were observed at follow-up (20.7% of all visitors). 44% more older adults were observed at follow-up in the NOS with the renovated benches.  Sedentary  behavior was more prevalent at follow-up (47.4%) compared to baseline (27.8%), walking activity became less prevalent with 65.6% of observed older adults walking at baseline, whereas only 45.6% walking at follow-up. |  |

*Note*. 6MWT = Six-Minute Walk Test. FSST = Four Square Step Test. IPAQ-SF = the international physical activity questionnaire. ISSB = Inventory of Social Supportive Behaviors. LSNS = Lubben Social Network Scale. LSNS-6 = 6-item Lubben Social Network Scale. LSNS-R = Lubben Social Network Scale Revised. M = mean. MSPSS = Multidimensional Scale of Perceived Social Support. MVPA = moderate-to-vigorous physical activity. PA = physical activity. PASE = Physical Activity Scale for the Elderly. SD = standard deviation. SF-36 = Short Form-36. SFT = senior fitness test. SPPB = Short Physical Performance Battery. SPS = Social Provisions Scale. SSQ = Social Support Questionnaire. TUG = Timed-Up and Go test. UCLA Loneliness Scale = The University of California, Los Angeles (UCLA) Loneliness Scale.
